# Knockdown of LMTK3 in the Endometrioid Adenocarcinoma Cell Line Ishikawa: Inhibition of Growth and Estrogen Receptor α

**DOI:** 10.3389/fonc.2021.692282

**Published:** 2021-10-20

**Authors:** Guiyang Cai, Wei Sun, Fangfang Bi, Dandan Wang, Qing Yang

**Affiliations:** ^1^ Department of Obstetrics and Gynecology, Shengjing Hospital of China Medical University, Shenyang, China; ^2^ Department of Key Laboratory of Cell Biology, Ministry of Public Health and Key Laboratory of Medical Cell Biology, China Medical University, Shenyang, China

**Keywords:** LMTK3, endometrioid adenocarcinoma, ERα, proliferation, cell cycle, apoptosis

## Abstract

**Objective:**

The curative effect of high-efficiency progesterone and other therapeutic drugs for endometrioid adenocarcinoma patients with preservation of reproductive capacity has not been satisfactory so far. Novel therapeutic drugs need to be explored.

**Methods:**

We investigated the cytoplastic and nuclear expression levels of LMTK3 between endometrioid adenocarcinoma tissues and adjacent endometrial tissues by immunohistochemistry. We detected the effects of LMTK3 on cell viability of Ishikawa cells by CCK-8. We detected the effects of LMTK3 on cell cycle and apoptosis of Ishikawa cells by flow cytometry. We also detected the effects of LMTK3 knockdown on mRNA and protein levels of ERα by qRT-PCR and western blotting, respectively. We also used the cBioPortal online database to analyze the coexpression of *LMTK3* and *ESR1* in 1647 UCEC samples.

**Results:**

We used TMAs to identify that LMTK3 was mainly detected in the cytoplasm of endometrioid tissues, and cytoplasmic LMTK3 expression in endometrioid tissues was higher than that in adjacent endometrial tissues (*P* < 0.05). LMTK3 knockdown decreased the proliferation of Ishikawa cells through decreasing cell viability (*P* < 0.01), increasing G1 (*P* < 0.001) arrest, and promoting apoptosis (*P* < 0.01). There was a positive correlation between the mRNA expression levels of *LMTK3* and *ESR1* (Spearman: *P*=2.011e-5, R=0.13; Pearson: *P*=7.18e-8, R=0.17). Knockdown of LMTK3 also reduced the mRNA (*P* < 0.001) and protein (*P* < 0.001) levels of ERα.

**Conclusions:**

Inhibitors of LMTK3 may be a possible future treatment for ERα and LMTK3 highly expressed endometrioid adenocarcinoma following appropriate studies.

## Introduction

Uterine corpus endometrial carcinoma (UCEC) is the second most common malignant tumor of the female reproductive system in China (1) and the first in the United States; there were 65,620 estimated new cases and 12,590 estimated deaths in 2020 in the United States (2). The incidence rate of UCEC has been increasing by 1.3% per year with the increased age of first birth as well as the increased obesity (3). About 14% of UCEC cases are diagnosed in premenopausal women, 5% of whom are younger than 40 years, and the number is increasing. In March 2020, the national comprehensive cancer network (NCCN) published the “2020 NCCN clinical practice guidelines for uterine cancer (1st edition),” which clearly pointed out that regardless of the stage of UCEC, surgical treatment is mainly used, such as hysterectomy, bilateral oviduct oophorectomy, and pelvic lymph node dissection, and fertility preserving treatment is only applicable to patients with endometrioid adenocarcinoma (EEC) (4). However, fertility preservation is so important for younger patients with EEC who have fertility requirements (5). At present, fertility preservation is mainly treated by progesterone, medroxyprogesterone acetate, and levonorgestrel releasing intrauterine system (5, 6). The current status of progesterone treatment is as follows: relatively low efficiency, high recurrence rate, and low pregnancy rate and live birth rate after progesterone treatment. A meta-analysis of 27 studies showed that the complete remission rate after oral progesterone treatment was 76.3%, the complete remission rate after intrauterine progesterone treatment was 72.9%, and the pregnancy rate after oral progesterone treatment was 52.1% (7). A meta-analysis of 32 studies also showed that the recurrence rate of progesterone treatment was 41%, and the recurrence rate continued to increase at least within 5 years; the live birth rate after progesterone treatment was 28% (8). Another retrospective study mentioned that the recurrence rate after oral progesterone treatment was 81.8%, and the pregnancy rate after high-dose progesterone treatment was only 6.3% (9). In addition, there are many adverse reactions of progesterone therapy, such as liver function damage, body mass index (BMI) increase, thrombophlebitis, headache, sleep disorders, and mood changes. Some patients who had poor compliance even had to terminate treatments. Therefore, looking for new therapeutic targets and developing highly effective molecular targeted drugs with less side effects have become the focus of fertility preservation research and the key to success of fertility preserving therapy for EEC patients.

Lemur tyrosine kinase 3 (LMTK3, also known as LMR3, TYKLM3, and KIAA1883) is an oncogenic kinase associated with proliferation in different types of cancer [breast (10), lung (11), stomach (12), and colon (13)]. In breast cancer MCF-7 cells, LMTK3 knockdown also has a cell growth inhibition function and decreases the mRNA and protein levels of estrogen receptor α (ERα) (14), and Guanli Hu et al. had reported that knockdown of ERα could inhibit the cell viability of Ishikawa cells at 48 h and increase G1 cell cycle arrest through upregulating P21 and inhibiting Cyclin D1 expression (15). However, whether knockdown of LMTK3 inhibits cell growth and ERα expression in the endometrioid adenocarcinoma cell line Ishikawa with a high expression of ERα (16) is still not known. In this paper, we found that knockdown of LMTK3 in Ishikawa did inhibit cell viability and increase G1 arrest and apoptosis, and also inhibited the mRNA and protein levels of ERα. We also used tissue microarrays (TMAs) and immunohistochemistry to find the phenomenon that the expression level of LMTK3 did have differences in EEC tissues, which was predominantly expressed in the cytoplasm of EEC tissues. Since EEC accounts for more than 80% of the newly diagnosed UCEC cases, in general, which is ERα positive (17, 18), estrogens control a series of genes involved in proliferation mainly mediated by ERα (15, 19); our results also identified that knockdown of LMTK3 in Ishikawa can inhibit cell growth and ERα expression, which may provide a new drug target for the fertility preserving therapy of EEC with a high expression of ERα and LMTK3 in the future.

## Methods

### Cell Lines and Antibodies

Ishikawa, well-differentiated endometrioid adenocarcinoma cells, obtained from Shanghai Cell Bank of Chinese Academy of Sciences, which were cultured in RPMI 1640 supplemented with 10% FBS and 1% penicillin/streptomycin, were incubated at 37°C with 5% CO_2_. The following antibodies were used: ERα rabbit monoclonal (#8644, Cell Signaling) and LMTK3 mouse monoclonal (#M06A, Abnova and #110516, Abcam).

### cBioPortal Database Analysis

We used cBioPortal (http://www.cbioportal.org/) (20, 21), an online database, using 1,647 endometrial cancer samples to analyze the coexpression of *LMTK3* and *ESR1*.

### Immunohistochemistry

TMAs consisting of 34 human EEC tissues were purchased from Outdo Biotech Co. Ltd. Immunohistochemical staining was performed using Biotin-Streptavidin HRP Detection Systems (Zhongshan Golden Bridge) according to the manufacturer’s instructions. After deparaffinization and rehydration, TMA was pretreated using heat-mediated antigen retrieval with sodium citrate buffer (pH6.0) for 20 min. Then TMA was incubated with 10 µg/ml anti-LMTK3 rabbit polyclonal antibody at room temperature for 15 min. A goat antirabbit secondary antibody was used to detect the primary. All the tissue information on the TMA was scanned and imaged by an automatic digital slide scanner (3DHISTECH, Pannoramic MIDI) to form a document. This document was opened by pannoramic viewer software to magnify 1–400 times for observation and image capture and then was analyzed to obtain nuclear and cytoplasmic staining. LMTK3 immunoreactivity was mainly detected in the cytoplasm of endometrial carcinoma and adjacent tissues epithelial cells, and little in the nucleus to a variable degree. Cytoplasmic staining was scored based on intensity ranging from 0 to +3, where 0 = null, +1 = low, +2 = intermediate, and +3 = high level of staining intensity. The positive rate of staining was 0–100%. Cytoplasmic H-scores = intensity score x staining positive rate, which were as follows: low (1–80) and high (81–200).

### Cell Viability Assay

Cells were seeded in 96-well plates at 1×10^4^/well and then was transfected with a control siRNA, siLMTK3-1, siLMTK3-2, and siLMTK3-3 plasmids respectively generated by GeneChem Co. Ltd. Proliferation assay was performed using a CCK-8 reagent (Beyotime). The absorbance at 450 nm was detected after 24- and 72-h transfection. The experiment was repeated three times independently.

### Annexin V/PI Apoptosis Assay

After transfection for 48 h with a control siRNA and siLMTK3-3 plasmids, respectively, the cells were digested with 0.25% trypsin without EDTA. After digestion, the cells were collected and centrifuged at 1,500 rpm for 5 min. The supernatant was removed and resuspended with PBS. The cells were washed twice with PBS at 1,500 rpm for 5 min. Apoptosis detection kit (Beyotime) was used for detection. The cells were resuspended with 195 μl annexin V-FITC binding buffer, then added with 5 µl annexin V-FITC, and after being mixed well, 10 ul PI was added; the reaction was at room temperature without light at 20 min and then detected by flow cytometry (Beckmancoulter, cytoFLEX).

### Cell Cycle

After transfection for 48 h with a control siRNA and siLMTK3-3 plasmids, respectively, the cells were digested with 0.25% trypsin without EDTA. After digestion, the cells were collected and centrifuged at 1,000 rpm for 5 min. The supernatant was removed and resuspended with PBS. The cells were washed twice with PBS at 1,000 rpm for 5 min. The cells were resuspended with 100 µl PBS. About 700 µl of precooled 80% ethanol was added slowly to make the final concentration of ethanol of 70%. The cells were fixed at 4 °C for more than 4 h. The cells were washed twice with precooled PBS at 1,000 rpm for 5 min. About 100 µl RNase (50 µg/ml) was added at 37 °C for 30 min; 400 µl PI (50 µg/ml) was added and dyed at 4 °C without light for 30 min and was then detected by flow cytometry (Beckmancoulter, cytoFLEX).

### RNA Isolation and Quantitative qRT-PCR

Total RNA was isolated using RNAiso Plus (9108, Takara). Reverse transcription was performed using a PrimeScript™ RT reagent kit with a gDNA eraser (RR047, Takara). qRT-PCR analysis was performed on a QuantGene 9600 (Bioer) using primers for LMTK3 and ERα purchased from Sangon Biotech. The primers for *LMTK3* are as follows—forward: 5’-CAAGTGCTGTGGTTGTGTAATG-3’; reverse: 5’-CAGGCATCTTGTCGAGGATGG-3’. The primers for *ESR1* are as follows—forward: 5’-GGGAAGTATGGCTATGGAATCTG-3’; reverse: 5’-TGGCTGGACACATATAGTCGTT-3’.

### Western Blotting

Whole cell lysates were prepared using NP40 lysis buffer (50 mM Tris-HCl, pH 8.0, 150 mM NaCl, 10% (v/v) glycerol, 1% NP40, 5 mM dithiothreitol (DTT), 1 mM EDTA, 1 mM EGTA, 50 µM leupeptin, and 30 µg/ml aprotinin). These extracts were then centrifuged at 15,000 rpm for 15 min at 4°C. The bicinchoninic acid (BCA) protein assay (Pierce) was used to determine the protein concentration. Lysates were incubated in 6x sodium dodecyl sulfate (SDS) sample buffer (5 min, 100°C), subjected to 8% or 10% SDS-PAGE, and blotted on a nitrocellulose membrane (GE Healthcare). The membranes were then blocked in TBS containing 0.1% (v/v) Tween20 and 5% (w/v) non-fat milk for 1 h and incubated overnight with different antibodies. After being washed with TBS/Tween20 clearly, the membranes were incubated with goat antirabbit IgG or goat antimouse IgG (1:15,000 dilution) for 60 min. The immunocomplexes were detected using enhanced chemiluminescence (ECL) detection. The intensity of the bands was quantified using the Image J software (NIH, Bethesda, MD).

### Statistical Analysis

All statistical analysis was performed using the software packages SPSS 22.0 and GraphPad Prism 5.0. Our data were expressed as x ± SEM; the appropriate t test was applied to analyze the statistical significance between two groups. P < 0.05 was considered to have statistical significance.

## Results

### LMTK3 Mainly Exists in the Cytoplasm of Endometrioid Tissues and Has Significant Difference Between EEC Tumor and Para-Tumor Tissues

**Figure 1:**
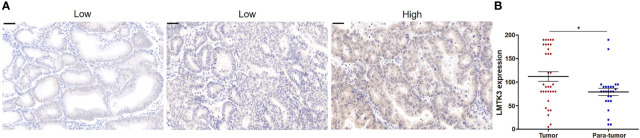
The cytoplastic expression of LMTK3 with significant difference between EEC tumor and para-tumor groups of tissue microarray. **(A)** Low and high staining of LMTK3 in endometrioid adenocarcinoma tissues; scale bar: 50 µM. **(B)** Scatter plot of cytoplastic LMTK3 expression with significant difference between EEC tumor and para-tumor groups, *P < 0.05.

We used TMAs to explore the LMTK3 expression between EEC and adjacent endometrial tissues, and the level of LMTK3 immunostaining analysis of TMA section revealed that LMTK3 was predominantly detected in the cytoplasm of endometrioid tissues with little in the nucleus of endometrioid tissues (**Figure 1A**). The cytoplasmic LMTK3 expression in endometrioid tissues was higher than that in adjacent endometrial tissues (**Figure 1B**); however, the nuclear LMTK3 expression did not have significant difference between endometrioid tissues and adjacent endometrial tissues (data not shown).

### Knockdown of LMTK3 Inhibits the Cell Viability of Ishikawa Cells

**Figure 2:**
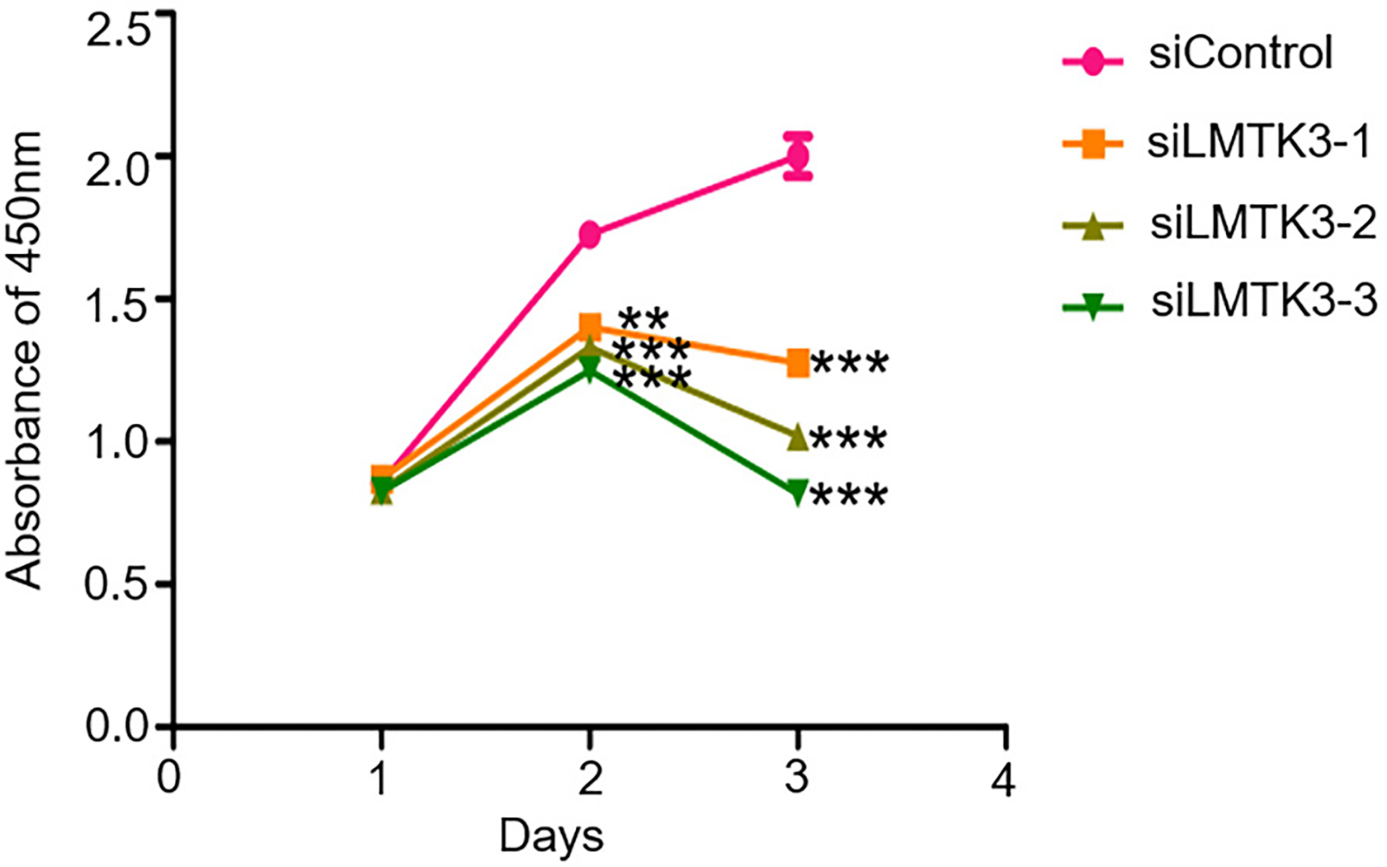
Effects of LMTK3 knockdown on cell viability in Ishikawa cells by CCK-8 (n=3, **P < 0.01, ***P < 0.001).

After 48-h transfection with a control siRNA, siLMTK3-1, siLMTK3-2, or siLMTK3-3 plasmid, the results showed that the A450 of the siRNA control group was 1.73 ± 0.03; however, the A450 of the three siLMTK3 groups were 1.40 ± 0.04, 1.33 ± 0.04, and 1.25 ± 0.04, respectively; the cell viability of the three siLMTK3 groups was lower than that of the siRNA control group (*P* < 0.01). After 72-h transfection with a control siRNA, siLMTK3-1, siLMTK3-2, or siLMTK3-3 plasmid, the A450 of the siRNA control group was 2.00 ± 0.07, the absorbance at 450 nm of the three siLMTK3 groups were 1.28 ± 0.04, 1.02 ± 0.05, and 0.82 ± 0.02, respectively, and the cell viability of the three siLMTK3 groups was also lower compared with the siRNA control group (*P* < 0.001) (**Figure 2**).

### Knockdown of LMTK3 Increases G1 Cell Cycle Arrest of Ishikawa Cells

**Figure 3:**
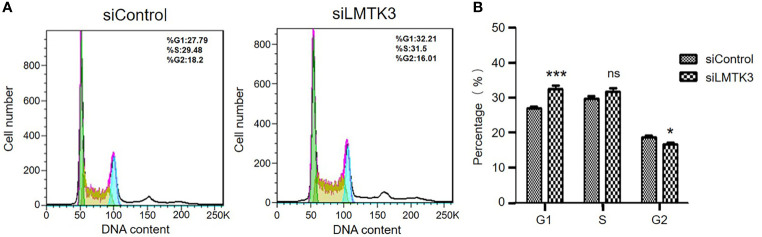
Effects of LMTK3 knockdown on cell cycle arrest in Ishikawa cells by flow cytometry. **(A)** Representative diagrams of LMTK3 knockdown on cell cycle arrest in Ishikawa cells. **(B)** Statistical histogram of LMTK3 knockdown on cell cycle arrest in Ishikawa (n=3, *P < 0.05, ***P < 0.001). ns represents no statistical significance).

After 48-h transfection with a control siRNA or siLMTK3 plasmid, the number of G1 phase cells of Ishikawa cells transfected with a control siRNA plasmid was 26.96 ± 0.40%, and that of Ishikawa cells transfected with plasmid siLMTK3 was increased to 32.53 ± 0.81% (*P*<0.001) (**Figures 3A, B**). The number of S phase cells of Ishikawa cells transfected with a control siRNA plasmid did not change compared with that of Ishikawa cells transfected with plasmid siLMTK3 after 48-h transfection (**Figures 3A, B**). The number of G2 phase cells of Ishikawa cells transfected with a control siRNA plasmid was 18.59 ± 0.49%, which is a little higher than that of Ishikawa cells transfected with plasmid siLMTK3 (16.65 ± 0.57%) after 48-h transfection (*P* < 0.05) (**Figures 3A, B**).

### Knockdown of LMTK3 Promotes the Early and Late Apoptosis of Ishikawa Cells

**Figure 4:**
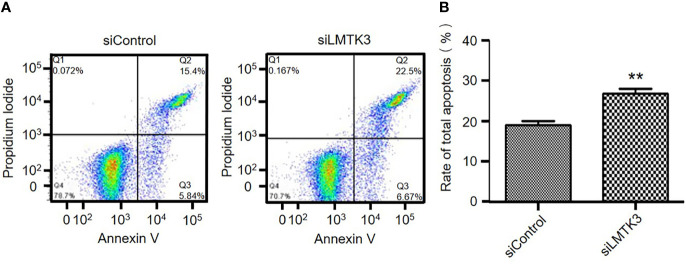
Effects of LMTK3 knockdown on apoptosis in Ishikawa cells by flow cytometry. **(A)** Representative diagrams of LMTK3 knockdown on apoptosis in Ishikawa cells. **(B)** Statistical histogram of LMTK3 knockdown on apoptosis in Ishikawa (n=3, **P < 0.01).

Because increasing apoptosis is another factor that has an inhibition effect on cell growth, after 48-h transfection with a control siRNA or siLMTK3 plasmid, we detected the apoptosis of Ishikawa cells. The results indicated that the early apoptosis rate of Ishikawa cells transfected with a control siRNA plasmid was only 4.31 ± 0.60%, and that of Ishikawa cells transfected with plasmid siLMTK3 was increased to 7.08 ± 0.61% (*P*<0.05) (**Figure 4A** and **Supplementary Figure 1A**). The late apoptosis rate of Ishikawa cells transfected with a control siRNA plasmid was only 14.58 ± 0.37%, and that of Ishikawa cells transfected with plasmid siLMTK3 was 19.75 ± 1.55%, which was higher than that of Ishikawa cells transfected with a control siRNA plasmid (*P*<0.05) (**Figure 4A** and **Supplementary Figure 1B**). As shown in **Figure 4B**, the total apoptosis rate of Ishikawa cells transfected with a control siRNA plasmid was only 18.89 ± 0.95%, and that of Ishikawa cells transfected with plasmid siLMTK3 was increased to 26.83 ± 1.10% (*P*<0.01). All above made it clear that LMTK3 knockdown increased both early and late apoptosis of Ishikawa cells compared with the transfection with a control siRNA plasmid.

### Knockdown of LMTK3 Reduces the mRNA and Protein Levels of ERα

**Figure 5:**
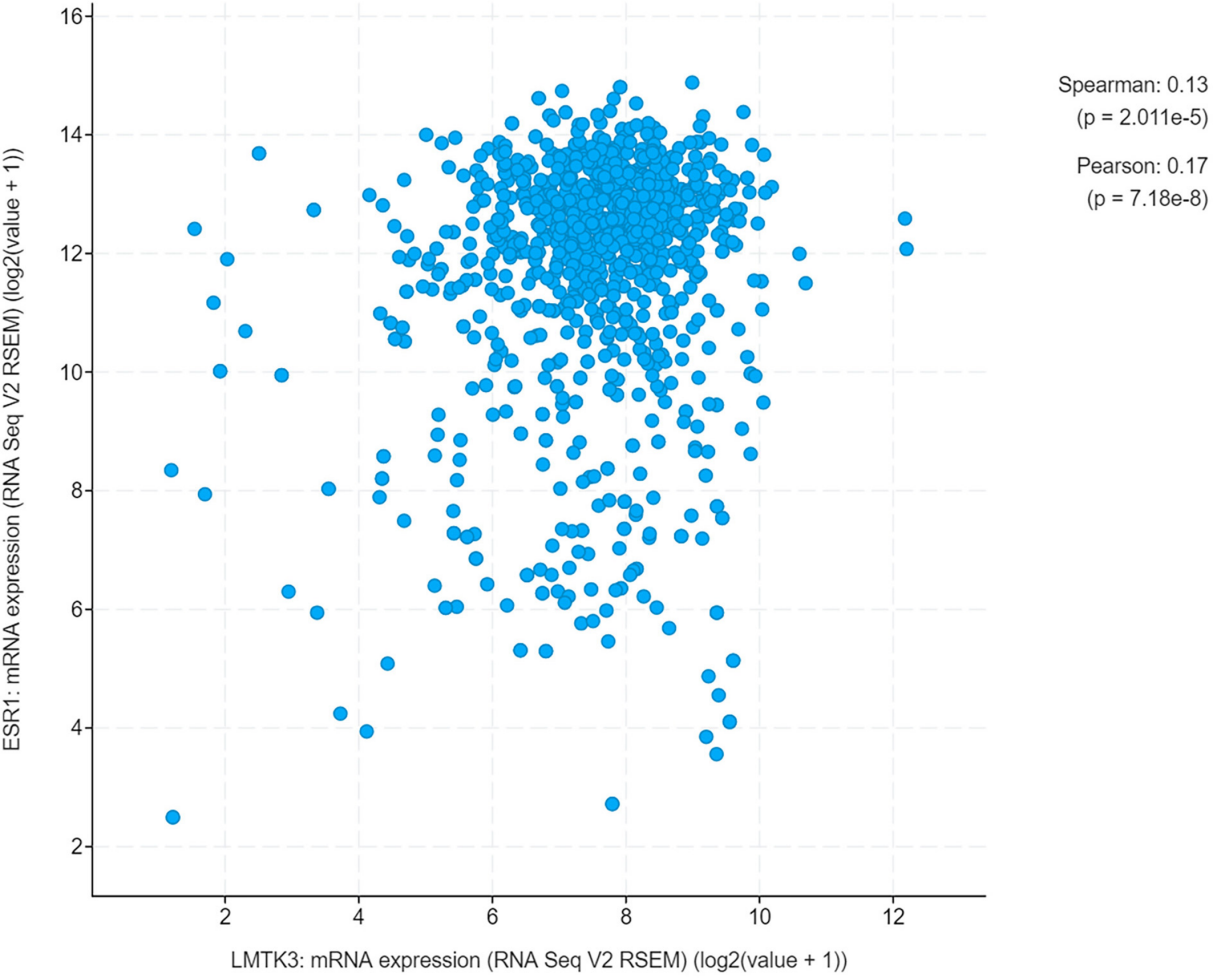
A positive correlation between the mRNA expression of *LMTK3* and *ESR1*.

**Figure 6:**
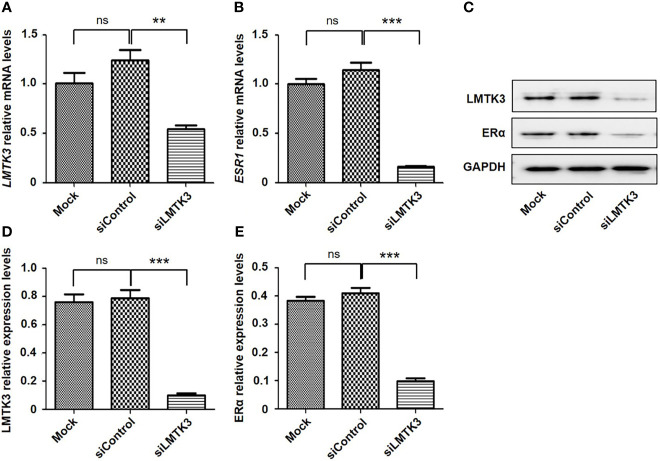
Knockdown of LMTK3 reduces the mRNA and protein levels of ERα. **(A, B)** Effects of LMTK3 knockdown on the mRNA level of ERα (n=3, **P < 0.01, ***P < 0.001), ns represents no statistical significance). **(C)** Representative image of LMTK3 knockdown on the protein level of ERα. **(D, E)** Effects of LMTK3 knockdown on the protein level of ERα (n=3, ***P < 0.001). siControl represents nontargeting siRNA. Mock represents nontreatment. ns represents no statistical significance).

We conducted an analysis of UCEC cases through cBioPortal, which included 1,647 UCEC cases. The results showed that there was a positive correlation between the mRNA expression levels of *LMTK3* and *ESR1* (Spearman: *P*=2.011e-5, R=0.13; Pearson: *P*=7.18e-8, R=0.17) (**Figure 5**). Therefore, we continued to explore the correlation between the above two genes at the cellular level. After 48-h transfection with a control siRNA or siLMTK3 plasmid, when the *LMTK3* relative mRNA level of the control siRNA group was 1.25 ± 0.10, and that of the siLMTK3 group was reduced to 0.55 ± 0.05 (*P* < 0.01) (**Figure 6A**), the *ESR1* relative mRNA level of the control siRNA group was 1.14 ± 0.08, and that of the siLMTK3 group was also reduced to 0.29 ± 0.03 (*P* < 0.001) (**Figure 6B**). After 48-h transfection with a control siRNA or siLMTK3 plasmid, when the LMTK3 relative protein level of the control siRNA group was 0.79 ± 0.058, and that of the siLMTK3 group was reduced to 0.10 ± 0.015 (*P* < 0.001) (**Figures 6C, D**), the ERα, a protein coded by *ESR1*, relative protein level of the control siRNA group was 0.41 ± 0.019, and that of the siLMTK3 group was reduced significantly to 0.098 ± 0.010 (*P*<0.001) (**Figures 6C, E**).

## Discussion

In this paper, we used TMAs to identify that the cytoplasmic LMTK3 expression in endometrioid tissues was higher than that in para-tumor endometrial tissues. We also identified that LMTK3 knockdown decreased the cell growth of Ishikawa cells through decreasing cell viability, increasing G1 arrest, and promoting apoptosis. We also used bioinformatics methods to explore the positive correlation between the mRNA expression of *LMTK3* and *ESR1* in 1,647 UCEC samples, and finally we found that LMTK3 knockdown really reduced the mRNA and protein levels of ERα. In short, knockdown of LMTK3 inhibiting cell growth and ERα expression in a well-differentiated ERα-positive endometrioid adenocarcinoma Ishikawa cell line has been first identified. As Hu G et al. had reported that knockdown of ERα could inhibit the cell viability of Ishikawa cells at 48 h and increase G1 arrest, and the type, source, and culture environment of cells were consistent with us (15), we can infer that knockdown LMTK3 can inhibit the cell viability of Ishikawa cells at 48 h and increase G1 arrest through downregulating the mRNA and protein expression level of ERα. Of course, this may not be the only one mechanism of LMTK3 knockdown inhibiting cell growth in Ishikawa cells, since LMTK3 can bind *via* DDX5 to the pri-miRNA of miR-34a and miR-182, thereby sequestrating them from further miRNA maturation processing. Ectopic expression of miR-34a and miR-182 in LMTK3-overexpressing breast cancer cell lines inhibited cell proliferation through directly binding to the 3’UTR of LMTK3 mRNA and consequently inhibiting both its stability and translation (22). LMTK3 also functioned at distal regions in tethering the chromatin to the nuclear periphery resulting in H3K9me3 modification and tumor suppressor like-gene silencing (23); therefore, knockdown of LMTK3 may also inhibit the growth of Ishikawa cells through other ERα independent mechanisms, which needs further studies.

We also identified for the first time that LMTK3 was predominantly detected in the cytoplasm of endometrioid adenocarcinoma tissues, as Stebbing J et al. found that LMTK3 was mainly expressed in the nucleus of breast cancer tissues with variable cytoplastic staining (24), which suggests that the role of LMTK3 in EEC and breast cancer may have similarities and differences. In this study, we did find that LMTK3 knockdown could inhibit cell viability, enhance G1 cell cycle arrest, promote apoptosis, and reduce the mRNA and protein levels of ERα in both ERα-positive EEC Ishikawa and breast cancer MCF-7 cells. We also found a difference that knockdown of LMTK3 was more likely to increase early apoptosis in MCF-7 cells compared with Ishikawa cells (14), the mechanism of which needs to be further explored in the future.

Although it has not been confirmed that LMTK3 is highly expressed in EEC tissues compared with normal tissues due to a small sample size, we also first discovered the phenomenon that LMTK3 did have different expression levels in endometrioid adenocarcinoma tissues, since knockdown of LMTK3 inhibiting cell growth and ERα expression in a well-differentiated ERα-positive endometrioid adenocarcinoma cell line Ishikawa has been confirmed in this paper; it was also reported that targeting LMTK3 may be a possible future treatment for bladder cancer (25), KIT-mutant gastrointestinal stromal tumor and subsets of melanoma (26), thyroid cancer (27), gastric cancer (12), prostate cancer (28), and estrogen positive breast cancer (10), which suggests that LMTK3 inhibitors may be a possible future treatment for ERα and LMTK3 highly expressed EEC following appropriate studies.

## Data Availability Statement

The original contributions presented in the study are included in the article/**Supplementary Material**. Further inquiries can be directed to the corresponding author.

## Author Contributions

WS and GC were responsible for confirming the topic. GC was responsible for writing the first draft of this article. FB, DW, and QY contributed to further editing and polishing the manuscript. All authors contributed to the article and approved the submitted version.

## Funding

The work was funded by the science and technology research project of education department of Liaoning province (No.LK201632), the National Natural Science Foundation of China (No.81872125), and Outstanding Scientific Fund of Shengjing Hospital (NO.201704).

## Conflict of Interest

The authors declare that the research was conducted in the absence of any commercial or financial relationships that could be construed as a potential conflict of interest.

## Publisher’s Note

All claims expressed in this article are solely those of the authors and do not necessarily represent those of their affiliated organizations, or those of the publisher, the editors and the reviewers. Any product that may be evaluated in this article, or claim that may be made by its manufacturer, is not guaranteed or endorsed by the publisher.

## Supplementary Material

Supplementary Figure 1 Statistical histogram of LMTK3 knockdown on early and late apoptosis in Ishikawa. **(A)** Statistical histogram of LMTK3 knockdown on early apoptosis in Ishikawa (n=3, **P* < 0.05). **(B)** Statistical histogram of LMTK3 knockdown on late apoptosis in Ishikawa (n=3, **P* < 0.05).
