# Supplementary figures and images for: Knockdown of LMTK3 in the Endometrioid Adenocarcinoma Cell Line Ishikawa: Inhibition of Growth and Estrogen Receptor α
Source: Front Oncol. 2021 Oct 20;11:692282. doi: 10.3389/fonc.2021.692282 (PMC8564183; doi:10.3389/fonc.2021.692282)

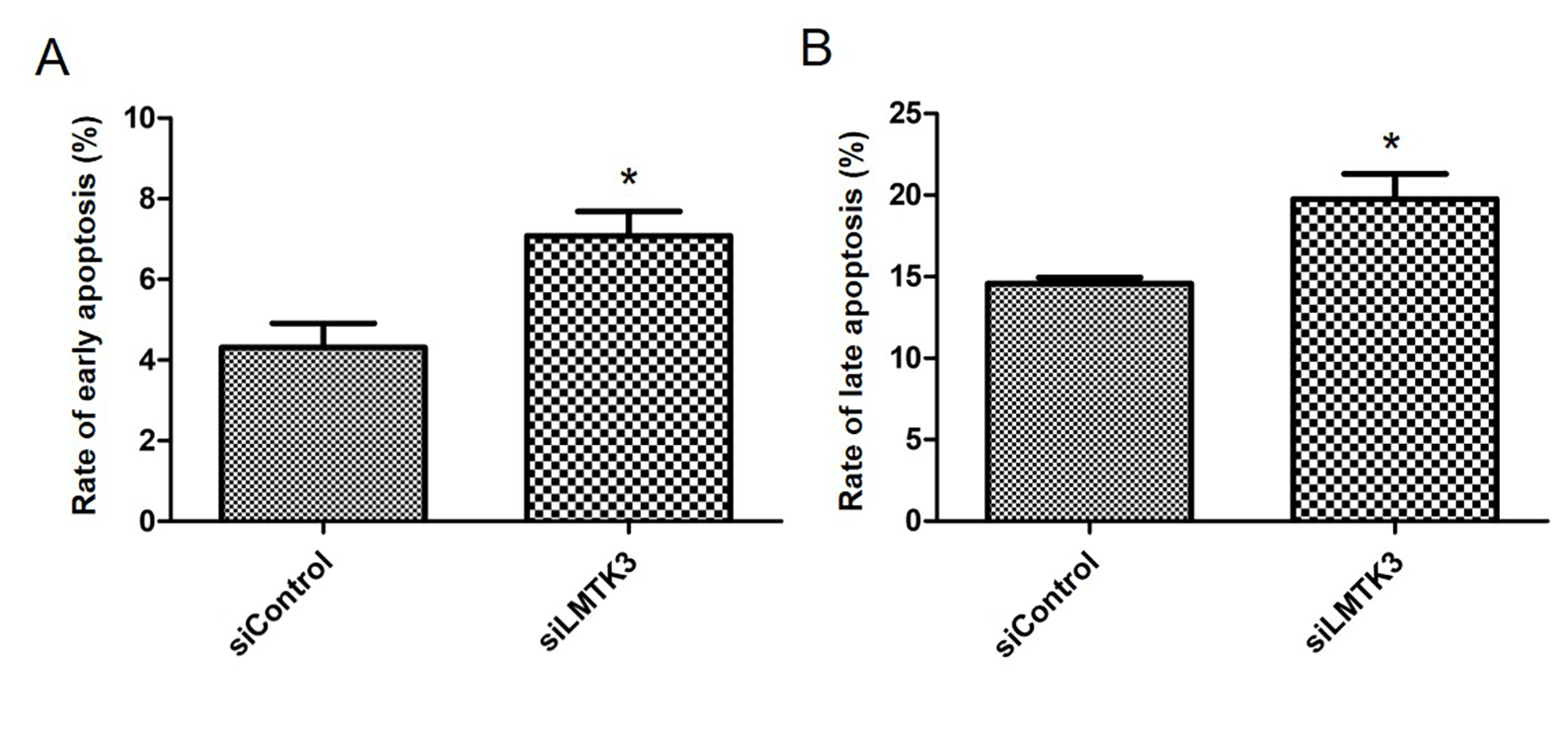

Supplement: Supplementary Figure 1 — Statistical histogram of LMTK3 knockdown on early and late apoptosis in Ishikawa. (A) Statistical histogram of LMTK3 knockdown on early apoptosis in Ishikawa (n=3, *P < 0.05). (B) Statistical histogram of LMTK3 knockdown on late apoptosis in Ishikawa (n=3, *P < 0.05). [file Image_1.tif]

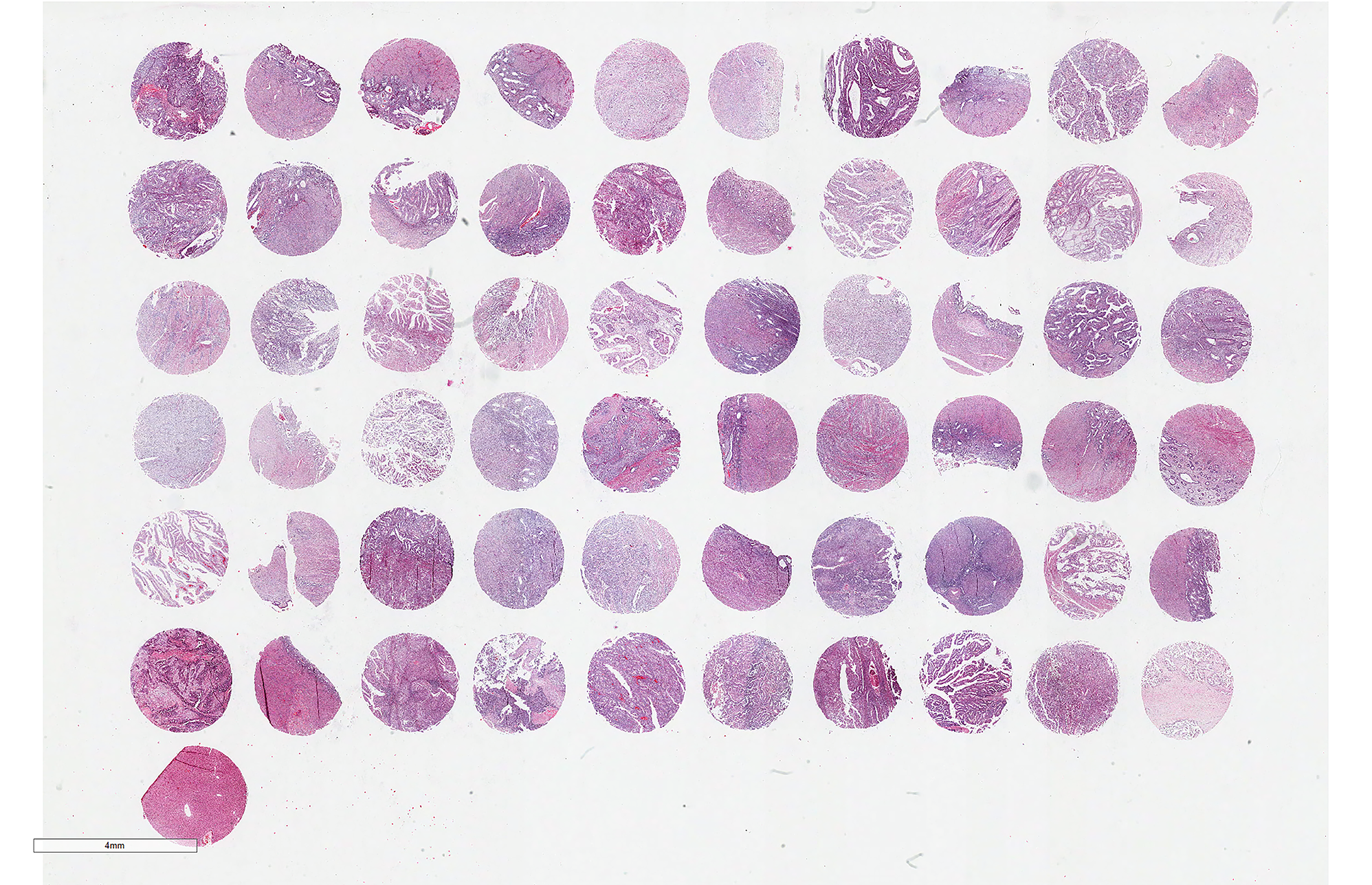

Supplement: Supplementary file 2 [file Image_2.tif]
